# Supplementary material for: Process bigraphs and the architecture of compositional systems biology
Source: ArXiv. 2026 Aug 14:arXiv:2512.23754v2. Preprint. [Version 2] (PMC13640864)
Supplement: Supplementary file 1 [file NIHPP2512.23754v2-supplement-1.pdf]

Supporting information

S1 Text. Formal framework specification (Supplement 1). Technical underpinnings of the Process Bigraph framework. (i) overview of the Vivarium 2.0 software suite; (ii) schema-based semantics – paths, schema trees and state trees, the type engine and type registry; (iii) textual representations of a process bigraph; (iv) links, ports, and the derived wiring map; (v) processes and *delta semantics* – typed function signatures, type-directed update operators; (vi) the tuple definition $B = (\Sigma, x, R_T, R_L)$ of a process bigraph; (vii) composites, their `interface` and `bridge`, and hierarchical composition; (viii) orchestration – discrete event scheduling, update reconciliation, workflows, and the large-scale orchestration; (ix) emitters and result capture; (x) execution protocols.

S2 Text. Spatio-Flux worked example (Supplement 2). A concrete Spatio-Flux instantiation of the Process Bigraph framework, mirroring the abstract semantics of S1 Text with executable process bigraphs. (i) Spatio-Flux schema-level types (fields, particles, `conc_counts`, complex particle records); (ii) typed process and step definitions for field dynamics (diffusion/advection), stochastic and deterministic particle movement, and particle–field metabolic coupling; (iii) composite documents and wiring diagrams for the multiscale examples used in the main text; (iv) a matched comparison between Spatio-Flux and the COMETS Java backend (via `cometspy` [26]); (v) runtime decomposition and wall-clock scaling results for local and distributed execution modes.
